# Prediction of prognosis of patients with lung cancer in combination with the immune score

**DOI:** 10.1042/BSR20203431

**Published:** 2021-05-14

**Authors:** Ke Han, Kun Qian, Teng Zhao, Xing Sheng Liu, Yi Zhang

**Affiliations:** Xuan Wu Hospital of Capital Medical University, Beijing 100053, China

**Keywords:** Immune score, Lung cancer, nomogram, prognosis

## Abstract

Purpose: The host’s immune response to malignant tumor is fundamental to tumorigenesis and tumor development. The immune score is currently used to assess prognosis and to guide immunotherapy; however, its association with lung cancer prognosis is not clear.

Methods: Clinical features and immune score data of lung cancer patients from The Cancer Genome Atlas were obtained to build a clinical prognosis nomogram. The model’s accuracy was verified by calibration curves.

Results: In total, 1005 patients with lung cancer were included. Patients were divided into three groups according to low, medium, and high immune scores. Compared with patients in the low immune score group, the disease-free survival (DFS) of patients in medium and high immune score groups was significantly longer; the hazard ratio (HR) and 95% confidence interval (95% CI) were 0.77 [0.60–0.99] and 0.74 [0.60–0.91], respectively. The overall survival (OS) of patients in the medium and high immune score groups was significantly longer than in the low immune score group; the HR and 95% CI were 0.74 [0.57–0.96] and 0.69 [0.55–0.88], respectively. A clinical prediction model was established to predict the survival prognosis. As verified by calibration curves, the model showed good predictive ability, especially for predicting 3-/5-year DFS and OS.

Conclusion: Patients with lung cancer with medium and high immune scores had longer DFS and OS than those in low immune score group. Patient prognosis can be effectively predicted by the clinical prediction model combining clinical features and immune score and was consistent with actual clinical outcomes.

## Introduction

Currently, the main prognostic indicators for patients with lung cancer are based on the tumor-lymph-node metastasis (TNM) staging system developed by the American Joint Committee on Cancer/Union for International Cancer Control (AJCC/UICC). This system provides exhaustive guidelines for the prognosis and treatment of patients with cancer. However, in clinical practice, there is a marked difference in the survival prognosis among patients having the same TNM stage. The disease development of some patients with advanced lung cancer may remain stable for several years [1,2]. In addition, approximately 10–25% of patients with TNM I/II tumors, who received prompt radical surgical treatment and had no lymph nodes or distant metastases according to pathological findings, experienced cancer recurrence and rapid progression or even death [2,3]. The reason for these differences is that the TNM assessment system is based solely on the biological behavior of the tumor, without considering the immune response of the host. Indeed, increasing evidence has indicated that cancer progression is greatly influenced by the host’s immune response [4–7]. The impact of the host’s immune response on the tumor is mainly manifested by the degree of infiltration of immune cells (mainly cytotoxic cells and memory T lymphocytes) in tumor tissues. Given the diversity of the immune microenvironment in colon cancer, Van den Eynde et al. reported on the key role played by cytotoxic cells and memory T lymphocytes in inhibiting the growth, invasion, and metastasis of primary tumors, and created a scoring system based on immune cell density for scoring immune microenvironments. The authors classified and quantitatively determined the lymphocyte populations infiltrating the tumor, and then used this information to predict the prognosis of patients with colon cancer, including patients with early-stage cancer [8]. The study by Minami et al. revealed the close correlation between immune infiltrating cells and the prognosis of patients with lung cancer. However, all these findings have not been converted into an effective tool for predicting the survival prognosis of patients with lung cancer [9].

Following a search of the literature, we learned that only a limited number of studies have been published on the assessment of the survival prognosis of patients with lung cancer based on the immune score. Thus, we established a clinical prediction model to estimate the survival prognosis of patients with lung cancer.

## Materials and methods

### Sample inclusion

The cases included in the present study were extracted from The Cancer Genome Atlas (TCGA), which currently contains data on over 200 types of cancer and the associated clinical information, and other information such as DNA methylation and RNA expression; it is the largest cancer genome analysis database in the world [10]. The sample information in the preent study was downloaded from the public website of TCGA. The contents downloaded included the identity (ID), sex, age, TNM status, clinical stage of tumor, pathological classification of tumor, disease-free survival (DFS), and overall survival (OS) of patients with lung cancer.

The immune score in the TCGA was calculated from tumor gene expression data. The overlapping data that constitute the immune signal was obtained from the comparison of the gene expression profile of normal hematopoietic tissues with that of other normal cells. The immune score was established to estimate the level of infiltrating immune cells and reflects the degree of infiltration of immune cells in tumor tissues. The basis and method of calculating the immune score in using TCGA datasets has been detailed in previous studies [11].

### Data preprocessing

**Figure 1:**
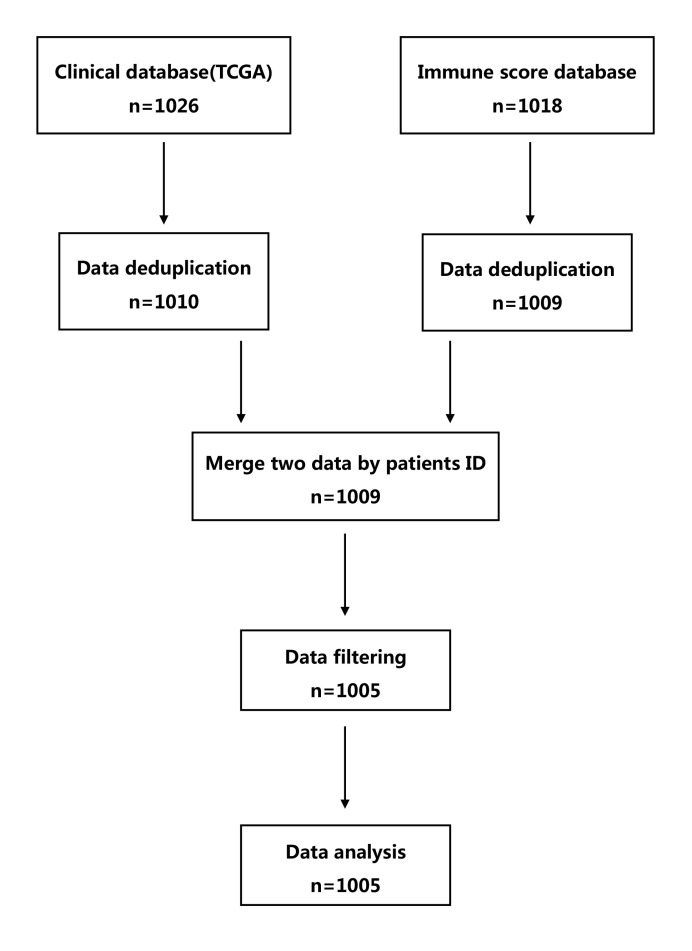
Flow chart Abbreviation: TCGA, The Cancer Genome Atlas.

Upon completion of the data retrieval, the data were screened first to remove duplicate cases and then cases with missing information. *R* software (Ver. 3.6.1) was used to match (integrate) the clinical characteristics and the immune score data according to the ID of patients with lung cancer, and to further analyze the integrated data. See Figure 1 for details on specific operating steps and the sample size at each stage.

### Statistical analysis

The main endpoint observation indicators in the present study were DFS and OS. OS was defined as death from any cause during the clinical course of a patient with lung cancer; while DFS was defined as the time before the recurrence of primary tumor. The software X‐tile 3.6.1 (School of Medicine, Yale University, U.S.A.) was used in combination with the immune score, the survival status, and OS of patients to determine the optimal cut-off value of the immune score and was used to divide the patients into three groups according to low, medium, and high immune scores. The chi-square test was adopted for categorical data. *R* software (Ver. 3.6.1) was used to establish the Kaplan–Meier survival curve. A univariate Cox proportional hazard regression model was used to analyze age, sex, TNM stage, pathological type of tumor and immune score, to identify independent predictors of DFS and OS, and to define the significant univariate variables to be included in the multivariate Cox proportional hazard regression model for analysis. Next, a prognosis nomogram was established. In this process, a bootstrap algorithm was adopted for internal verification. One thousand bootstrap experiments were conducted. To determine the consistency of the results obtained from the clinical prediction model and the actual clinical status of patients, the 3-/5-year DFS and OS calibration charts were drawn. All statistical tests were bilateral. *P*<0.05 was considered statistically significant.

## Results

### Clinical features of patients

A total of 1005 patients with lung cancer were included in the present study. The average age was 64.42 (range, 33–90) years and 731 patients (72.74%) were above age 60 years. There were 603 male patients (60.00%). In terms of TNM stage, 521 patients (51.84%) were at stage I, 284 patients (28.26%) at stage II, 167 patients (16.62%) at stage III, and 33 patients (3.29%) at stage IV. The average immune score was 723.74 (range, -1651.61 to 3286.67). The software X‐tile 3.6.1 (School of Medicine, Yale University, U.S.A.) was used to identify the most appropriate cut-off value of the immune score. The range of the low immune scores was -1651.6 to 698.1; the range of intermediate immune scores was 698.1 to 1246.3; and the range of high immune scores was: 1246.3 to 3286.7, which corresponded to 489 patients (48.66%) being allocated into the low immune score group, 213 patients (21.20%) into the intermediate immune score group, and 303 patients (30.15%) into the high immune score group. The median OS was 21.90 months (range, 0–238.11 months) and the DFS was 18.99 months (range, 0–238.11 months) (Table 1).

**Table 1:** Clinical features of patients with lung cancer

Clinical features	Total	Immune scores			X^2^	*P* value
		≤698.1	698.1-1246.3	>1246.3		
Sample sizes	1005	489	213	303	–	–
**Age (y)**					14.36	0.07
≤50	61 (6.07)	33	13	15		
51–60	213 (21.19)	114	34	65		
61–70	373 (37.11)	192	79	102		
71–80	310 (30.85)	134	73	103		
>80	48 (4.78)	16	14	18		
**Sex**					2.75	0.25
Male	603 (60.00)	301	132	170		
Female	402 (40.00)	188	81	133		
**TNM stage**					8.85	0.18
I	521 (51.84)	245	104	172		
II	284 (28.26)	143	56	85		
III	167 (16.62)	84	45	38		
IV	33 (3.28)	17	8	8		
**Pathological type**					4.02	0.13
Adenocarcinoma	510 (50.75)	233	111	166		
Squamous	495 (49.25)	256	102	137		

Abbreviation: TNM, tumor-lymph-node metastasis.

### Analysis of DFS and OS with univariate and multivariate Cox proportional hazard regression models

**Figure 2:**
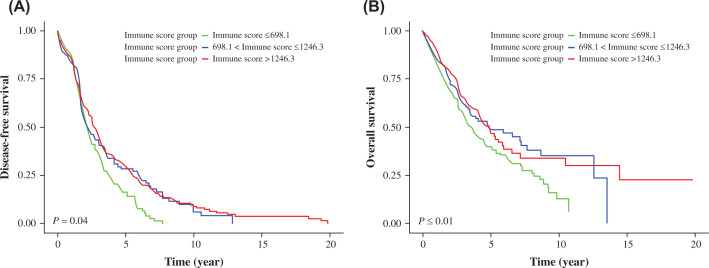
Correlation between Immune score and DFS and OS of patients with lung cancer described by Kaplan-Meier Curves Kaplan–Meier curves showing the relationship between immune score and DFS (**A**) and OS (**B**) in patients with lung cancer. Red line represents high immune score subgroup (>1246.3). Blue line represents medium immune score subgroup (698.1–1246.3). Green line represents low immune score subgroup (≤698.1). The *P*=0.04 at DFS and *P*≤0.01 at OS are presented. Abbreviations: DFS, disease-free survival; OS, overall survival.

The results of univariate Cox proportional hazard regression model showed that the DFS of the medium and high immune score groups was significantly longer than that of the low immune score group. The hazard ratio (HR) for the medium immune score group was 0.77 and 95% CI [0.60–0.99], *P*=0.04; while the HR for the high immune score group was 0.74 and 95% CI [0.60–0.91], *P*<0.01. Kaplan–Meier survival curves (Figure 2) indicated that there was a significant difference among different groups when stratified by age, sex, TNM stage, or pathological types. According to the results of the univariate analysis for OS, compared with TNM stage I patients, the survival prognosis of patients at stages II, III, and IV was significantly poorer. The HR and 95% CIs were as follows for the TNM stage II 1.59 [1.25–2.01]; TNM stage III 2.21 [1.71–2.85]; and TNM stage IV 3.06 [1.94–4.83] groups, respectively (for all *P*<0.01) (Table 2).

**Table 2:** Univariate cox proportional hazard regression analysis of DFS and OS of patients with lung cancer based on clinical features and immune score

Characteristics	Total	DFS				Total	OS			
		Nonrelapse	Relapse	HR (95%CI)	*P* value		Survival	Deadth	HR (95%CI)	*P* value
**Age (year)**										
≤50	49	19	30	1.00		61	37	24	1.00	
51–60	182	62	120	1.11 (0.74–1.67)	0.62	213	143	70	0.93 (0.57–1.50)	0.75
61–70	298	121	177	1.05 (0.71–1.56)	0.80	373	235	138	0.97 (0.62–1.53)	0.89
71–80	232	100	132	0.99 (0.66–1.48)	0.96	310	164	146	1.21 (0.77–1.91)	0.40
>80	35	11	24	1.39 (0.80–2.40)	0.24	48	26	22	1.57 (0.86–2.87)	0.14
**Sex**										
Male	462	181	281	1.00		603	350	253	1.00	
Female	334	131	203	1.05 (0.88–1.26)	0.57	402	256	146	0.87 (0.71–1.06)	0.16
**TNM stage**										
I	424	136	288	1.00		521	352	169	1.00	
II	231	104	127	0.98 (0.79–1.20)	0.82	284	165	119	1.59 (1.25–2.01)	<0.01**
III	123	63	60	0.95 (0.72–1.26)	0.73	167	76	91	2.21 (1.71–2.85)	<0.01**
IV	18	9	9	1.31 (0.67–2.53)	0.43	33	12	21	3.06 (1.94–4.83)	<0.01**
**Pathological type**										
Adenocarcinoma	426	183	243	1.00		510	326	184	1.00	
Squamous	370	129	241	0.99 (0.83–1.18)	0.91	495	280	215	1.12 (0.92–1.36)	0.26
**Immune score**										
≤698.1	242	91	151	1.00		489	352	137	1.00	
698.1–1246.3	169	64	105	0.77 (0.60–0.99)	0.04*	213	136	77	0.74 (0.57–0.96)	0.02*
>1246.3	385	157	228	0.74 (0.60–0.91)	<0.01**	303	200	103	0.69 (0.55–0.88)	<0.01**
Sum up	796					1005				

Abbreviations: DFS, Disease-Free Survival; OS, Overall Survival; TNM, tumor-lymph-node metastasis. *P＜0.05; **P＜0.01.

**Figure 3:**
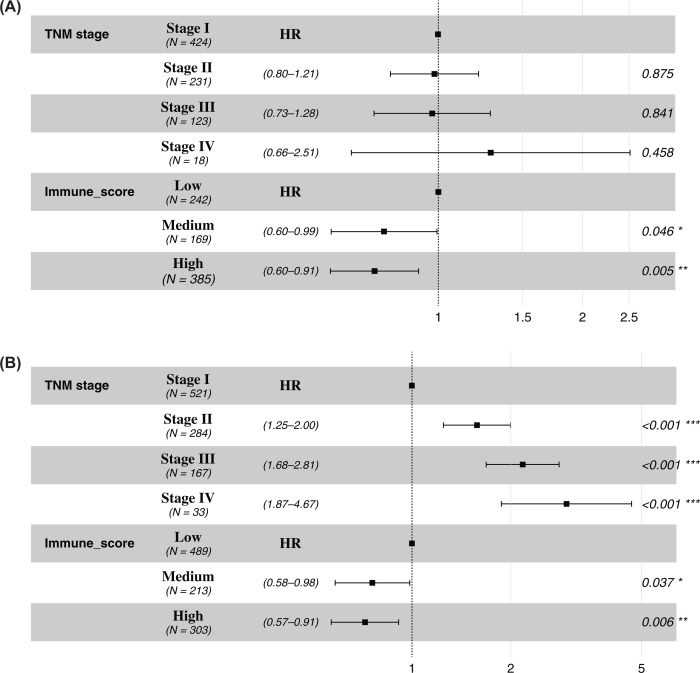
Multivariate Cox proportional hazard regression analysis of DFS and OS Forest plot of patients with lung cancer based on the TNM stage and the immune score Forest plots showed that DFS (**A**) and OS (**B**) were related to immune scores in patients with lung cancer. The results showed that high and medium immune score were associated with patients' DFS (*P*=0.04; *P*<0.01) and OS (*P*=0.04; *P*<0.01), and TNM stage was associated with patients' OS. Abbreviations: DFS, disease-free survival; OS, overall survival.

According to the results of the multivariate Cox proportional hazard regression analysis, significant difference in DFS remained between the low immune score group and the intermediate and high immune score groups, with HR and 95% CI of 0.77 [0.60–0.99] (*P*=0.04) and 0.74 [0.60–0.91] (*P*<0.01), respectively. The TNM groups showed no statistically significant differences. The HR and 95% CI for the TNM II, III, and IV stage groups were 0.98 [0.80–1.21] (*P*=0.88), 0.95 [0.73–1.28] (*P*=0.84), and 1.31 [0.66–2.51] (*P*=0.46), respectively. Our findings also showed that there was a significant difference in the OS among the different TNM and immune score groups, with HR and 95% CI for the TNM stage II, III, and IV groups of 1.58 [1.25–2.00] (*P*<0.01), 2.17 [1.68–2.81] (*P*<0.01), and 2.96 [1.87–4.67] (*P*<0.01), respectively. There was also a significant difference in the OS between the low immune score group and the medium and high immune score groups; the HR and 95% CI were respectively, 0.76 [0.58–0.98] (*P*=0.04) and 0.72 [0.57–0.91] (*P*<0.01) (Figure 3).

### Prognosis nomogram

**Figure 4:**
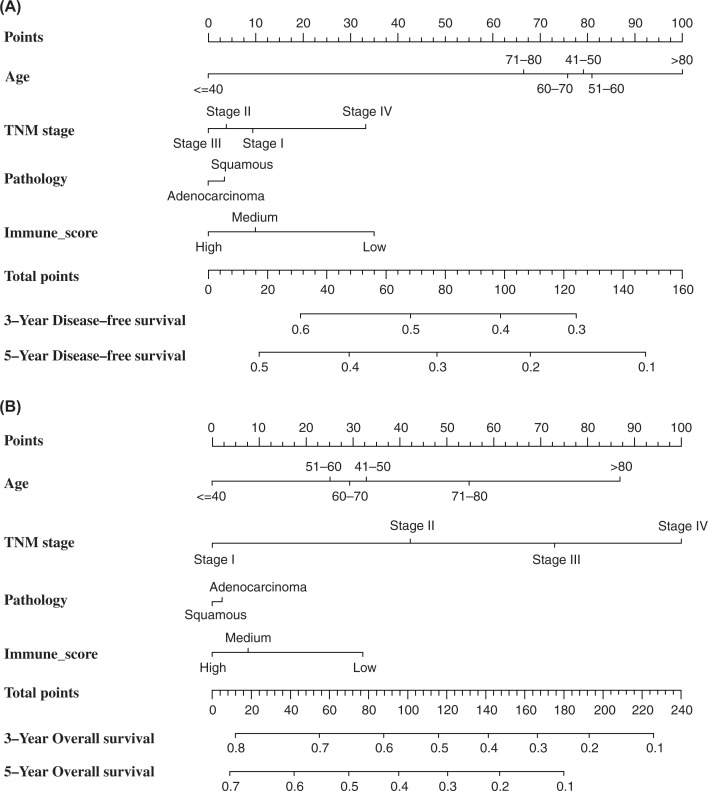
Prognosis Nomogram based on clinical features and immune score The Prognosis Nomogram predicted the 3-/5-year DFS (**A**) and OS (**B**) in patients with lung cancer. The points axis represents a value corresponding to a score of each factor (i.e., age, TNM stage, pathology, and immune score). The total points axis represents a value corresponding to total score of all factors. Survival axis represents the likelihood of 3- and 5-year survival according to total score. Abbreviations: DFS, disease-free survival; OS, overall survival.

**Figure 5:**
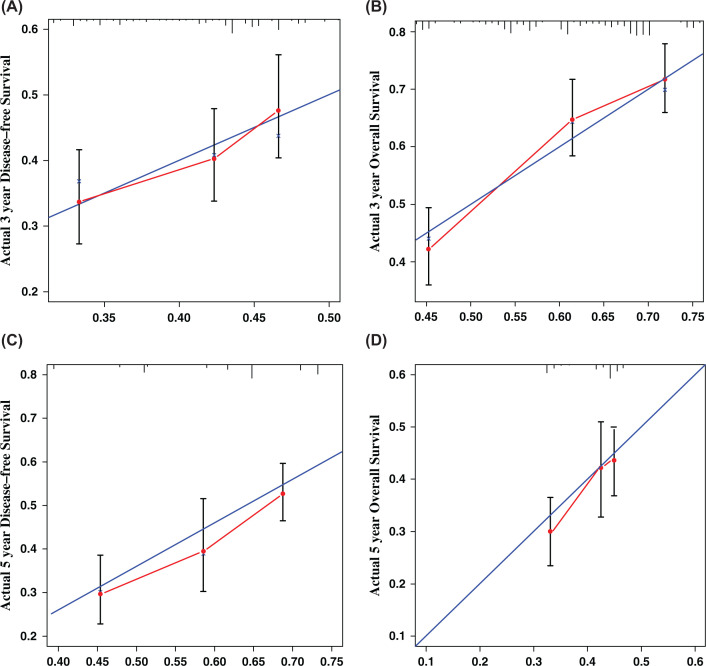
Three and five-year DFS and OS calibration curves for lung cancer Calibration curve was used to evaluate the predictive ability of the model for DFS (**A** and** C**) and OS (**B** and** D**) in patients with lung cancer. The *X*-axis represents nomogram-predicted probability of 3- and 5-year DFS or OS. *Y*-axis represents actual 3- and 5-year DFS or OS. Abbreviations: DFS, disease-free survival; OS, overall survival.

A clinical prognosis nomogram was established combining all the independent factors that we found were able to significantly predict the prognosis of patients with lung cancer (Figure 4). The 3-/5-year DFS and OS calibration charts were drawn to determine the accuracy of the clinical prediction model (Figure 5), which showed that the clinical prediction model is quite consistent with the actual patient outcomes. At the same time, we can see from the figure that compared with 3-year DFS and OS calibration charts, the prediction ability of 5-year DFS and OS calibration charts has decreased.

## Discussion

Lung cancer is a common malignant tumor with a complicated pathogenesis and high mortality that severely threatens public health worldwide. Currently, the TNM stage is considered an optimal indicator to estimate the prognosis of patients with lung cancer [12]. Considering the limitations of TNM staging, which is based only on the biological behavior of the tumor, continued in-depth studies on the tumor microenvironments in recent years have led to the hypothesis that a patient’s own immune system also plays an important role in the occurrence, invasion, and metastasis of tumors. Normal cells in the tumor microenvironment mainly include infiltrating stromal cells and immune cells, which affect the biological behavior of the tumor and regulate the signaling molecules in the tumor microenvironment [11,13]. Thus, the influence of the host’s infiltrating immune cells and the degree of autoimmunity of the tumor have received increasing attention and probably have an important impact on the prognosis of tumor patients. Increasing evidence indicates that the massive infiltration of intracellular cytotoxic T lymphocytes (CD8^+^) and memory T cells (CD4^+^) in lung cancer is closely correlated to the good prognosis of patients with lung cancer [13,14]. CD8^+^ T cells are the main effector cells, while CD4^+^ T cells can induce and activate CD8^+^ T cells in the tumor microenvironment. Studies have shown that such T lymphocytes play a role in inhibiting the growth, invasion, and metastasis of several tumors, such as lung, ovarian, and rectal cancers [4,15,16]. Further knowledge regarding tumor-associated normal cells in tumor tissues has deepened the understanding of the biological behavior of primary tumor and the host’s immune response and has provided new ideas for the treatment of tumor. Some researchers have proposed to use the immune score as an immune biomarker to guide the clinical immunotherapy of tumors [17,18]. Meanwhile, a more valuable clinical prediction model should be established in combination with the immune score to help clinicians assess the survival prognosis of tumor patients.

In the present study, we evaluated the prognostic value of immune score in patients with lung cancer. Univariate and multivariate analysis of OS and DFS showed that both moderate and high immune scores were significantly associated with good prognosis. In addition, we integrated all the clinicopathological factors to construct the nomogram survival prediction model for patients with clinical nomogram lung cancer.

The immune score in the present study was defined using a TCGA dataset and adopted a novel assessment method based on the transcription profiles of cancer samples to predict nontumor components in tumor cells and tumor tissues. Gene expression characteristics were used to infer the proportion of stromal and immune cells in tumor samples, and to identify the specific features associated with immune cell infiltration in tumor tissues. The immune score was calculated through enrichment analysis of single sample genes to predict the degree of infiltration of immune cells [19–23]. Such studies have been reported in several tumors, such as lung, breast, and ovarian cancers [24,25].

In the preent study, the immune score from TCGA was used to assess the prognosis of patients with lung cancer. Patients were divided into three groups according to the immune score: low, medium, and high immune score groups. We found that, compared with the low immune score group, the DFS and OS of patients with lung cancer in the medium and high immune score groups were significantly longer. A possible reason for this is as follows: the higher the immune score, the stronger the host’s autoimmunity to the tumor, the more immune cells can be mediated to enter the tumor microenvironment to play an antitumor role. The analysis of DFS with univariate and multivariate Cox proportional hazard regression models showed that, compared with immune score groups, the results in the TNM groups were positive, indicating the immune score may be superior to the TNM staging system in predicting the DFS of patients with lung cancer. This conclusion is of certain guiding significance in clinical practice. For patients with lung cancer at stage I/II according to the TNM staging system, if their immune score is low, they should be active during the treatment and should be closely followed-up to monitor disease development.

In order to predict the survival prognosis of patients with lung cancer more effectively and accurately, a survival prognosis model for patients with lung cancer was developed by combining previously commonly used clinical pathological features with the novel immune score. Moreover, the verification of this model showed that it had good predictive ability and could represent an effective reference for clinicians to better quantify the survival prognosis of patients with lung cancer. At the same time, we found that compared with 3-year DFS and OS calibration charts, the predictive power of 5-year DFS and OS calibration charts has declined. We believe that the reason is that human immunity is a changing process, and there is a time correlation. With the increase of age, changes in body function, gene expression and immunity of patients will change, leading to a decrease in the predictive power of this model.

Both patients and clinicians can achieve a more individualized survival prediction using this clinical prediction model and it may contribute to better predict a patient’s disease development. Further, the application of the immune score in patients with lung cancer will provide a basis for choosing a better treatment plan for clinical management. However, the present study has certain limitations mainly in the following aspects: the treatment conditions of patients with lung cancer included were unknown; thus, the effect of specific treatments on patient survival could not be determined. Therefore, it is necessary to collect additional relevant information from patients with lung cancer to be included in the study, so as to establish a more effective and accurate clinical prediction model.

## Conclusion

The immune score can be used as an effective indicator for predicting the survival prognosis of patients with lung cancer. The higher the immune score, the better prognosis of patients with lung cancer. In addition, the clinical prediction model established using the immune score can help clinicians assess the prognosis of patients with lung cancer more accurately, effectively, and conveniently and will assist in identifying patient subgroups requiring active adjuvant therapy, which underlines its certain clinical significance.

## Data Availability

All clinical pathological data of NSCLC patients can be downloaded directly from the cBioPortal database at: http://www.cbioportal.org/. All immune score data of NSCLC patients can be downloaded directly from the ESTIMATE database at: https://bioinformatics.mdanderson.org/estimate/. In addition, all codes used for data analysis are available from the corresponding author on responsible request.

## Abbreviations

95% CI: 95% confidence interval
DFS: disease-free survival
HR: hazard ratio
OS: overall survival
TNM: tumor-lymph-node metastasis
